# Oncogene c-Myc promotes epitranscriptome m^6^A reader YTHDF1 expression in colorectal cancer

**DOI:** 10.18632/oncotarget.23554

**Published:** 2017-12-21

**Authors:** Yujiro Nishizawa, Masamitsu Konno, Ayumu Asai, Jun Koseki, Koichi Kawamoto, Norikatsu Miyoshi, Hidekazu Takahashi, Naohiro Nishida, Naotsugu Haraguchi, Daisuke Sakai, Toshihiro Kudo, Taishi Hata, Chu Matsuda, Tsunekazu Mizushima, Taroh Satoh, Yuichiro Doki, Masaki Mori, Hideshi Ishii

**Affiliations:** ^1^ Department of Gastroenterological Surgery, Graduate School of Medicine, Osaka University, Suita, Osaka, Japan; ^2^ Department of Frontier Science for Cancer and Chemotherapy, Graduate School of Medicine, Osaka University, Suita, Osaka, Japan; ^3^ Department of Medical Data Science, Graduate School of Medicine, Osaka University, Suita, Osaka, Japan; ^4^ Department of Therapeutics for Inflammatory Bowel Diseases, Graduate School of Medicine, Osaka University, Suita, Osaka, Japan

**Keywords:** YTHDF1, colorectal cancer, c-MYC, proliferation, chemosensitivity

## Abstract

Recent studies that have emerged on the diversity of RNA modification in tumors suggest their eligibility as bona fide targets in diagnosis and drug discovery. N6-methyladenosine (m^6^A) was first reported and is most common in epitranscriptome modification of various RNAs. The YT521-B homology (YTH) domain family are representative m6A-binding proteins, but how the YTH domain family is involved in cancer remains to be clearly understood. Given that clinical sequence data in colorectal cancer indicate that overexpression of YTHDF1 is outstanding among other family members, we studied the role of Ythdf1 and the transcriptional control of YTHDF1. Immunostaining of Ythdf1 showed that its expression was associated with various malignant tumor behaviors, such as depth, lymph node metastasis, and poorer cancer stages. The study of patient survival indicated that patients with high Ythdf1 expression had significantly poorer overall survival. The results indicated that Ythdf1 expression is an independent prognostic factor of patients. The *in vitro* study showed that the knockdown of YTHDF1 resulted in the suppression of cancer proliferation and sensitization to the exposure of anticancer drugs such as fluorouracil and oxaliplatin. Importantly, the study upstream of the YTHDF1 gene indicated that an oncogenic transcription factor c-Myc was associated with YTHDF1 in both expression and chromatin immunoprecipitation data. Moreover, the knockdown experiments of c-Myc showed the inhibition of YTHDF1, supporting a notion of c-Myc-driven YTHDF1 axis significance. These data suggest that m6A reader Ythdf1 plays a significant role in colorectal cancer progression.

## INTRODUCTION

Globally, colorectal cancer (CRC) is the third most common cancer in men (746,000 cases annually, 10.0% of the total) and the second in women (614,000 cases, 9.2% of the total) [1]. Comprehensive genomic sequencing has been performed to evaluate genetic alterations in cancer, such as mutations in cancer-promoting genes or drug-resistant genes, although the direct sequence confers limited information in epigenetic modifications [2]. The study of cancer chronology supports the concept that multi-step processes are involved in tumor development, progression, and metastasis during carcinogenesis, and that genetic as well as epigenetic alterations define malignant tumor behavior [3]. In terms of epigenetic alterations, the mechanisms related to gene expression without a change in DNA base sequence [4], such as methylation of DNA and histone modifications, have been studied extensively by chromatin immunoprecipitation, followed by its related high-throughput sequencing, which indicated that information of epigenetic alterations can confer cancer diagnosis, druggable targets, and biomarkers [5].

On the other hand, RNA modifications remain to be understood. Although N6-methyladenosine (m^6^A) was first reported in a study of hepatoma cells about 40 years ago [6], the study of RNA methylation had not been much researched until recent years because it is difficult to detect due to instability and a short lifetime [7]. Nevertheless, recent studies have revealed that RNA modifications have been elucidated by the development of a specific antibody, and that the technology of mass spectrometry, as well as the identification of RNA methylases (writers of methylation) and demethylases (erasers) [8], also have made epitranscriptomics possible as druggable targets for many diseases, such as neurodegenerative disorders, developmental abnormalities [7], and cancer [9].

m^6^A is the most common modification among various RNA modifications [8]. It is known that m^6^A is present in about 7,000 mRNAs or noncoding RNAs in human cells [9]. m^6^A is involved in the control of various cell functions, such as circadian rhythm [10], meiosis [11], and stem cell development [12]. In addition to the above-mentioned “writers,” RNA methylates and “erasers,” demethylates, and a third group of “readers” bind to m^6^A and determine the fate of the modified mRNA [13].

The YT521-B homology (YTH) domain family (YTHDF1, 2, 3 and YTHDC1, 2) are known to be representative “reader” proteins. In humans, YTHDF1, 2, 3 function as m^6^A-specific binders in the cytoplasm. YTHDF2 specifically recognizes and destabilizes m^6^A-modified RNAs and re-localizes these RNAs to processing bodies, while YTHDF1 stimulates mRNA translation by interacting with translation initiation factors. To the best of our knowledge, the significance of the YTH domain family remains poorly understood.

Here, we studied the expression of the YTH domain family in The Cancer Genome Atlas (TCGA) database (https://cancergenome.nih.gov) and found that YTHDF1 is expressed in CRC portions compared with normal counterparts, while other YTH domain families showed little differences between cancer and normal portions. We then studied the functional role of Ythdf1 in cancer cell growth and chemoresistance as well as the transcriptional control of YTHDF1 transcript. Our study demonstrated the clinicopathological significance of the m^6^A reader Ythdf1, which is under the control of the oncogenic transcription factor c-Myc in CRC.

## RESULTS

### Ythdf1 is more highly expressed in CRC than normal tissues

**Figure 1:**
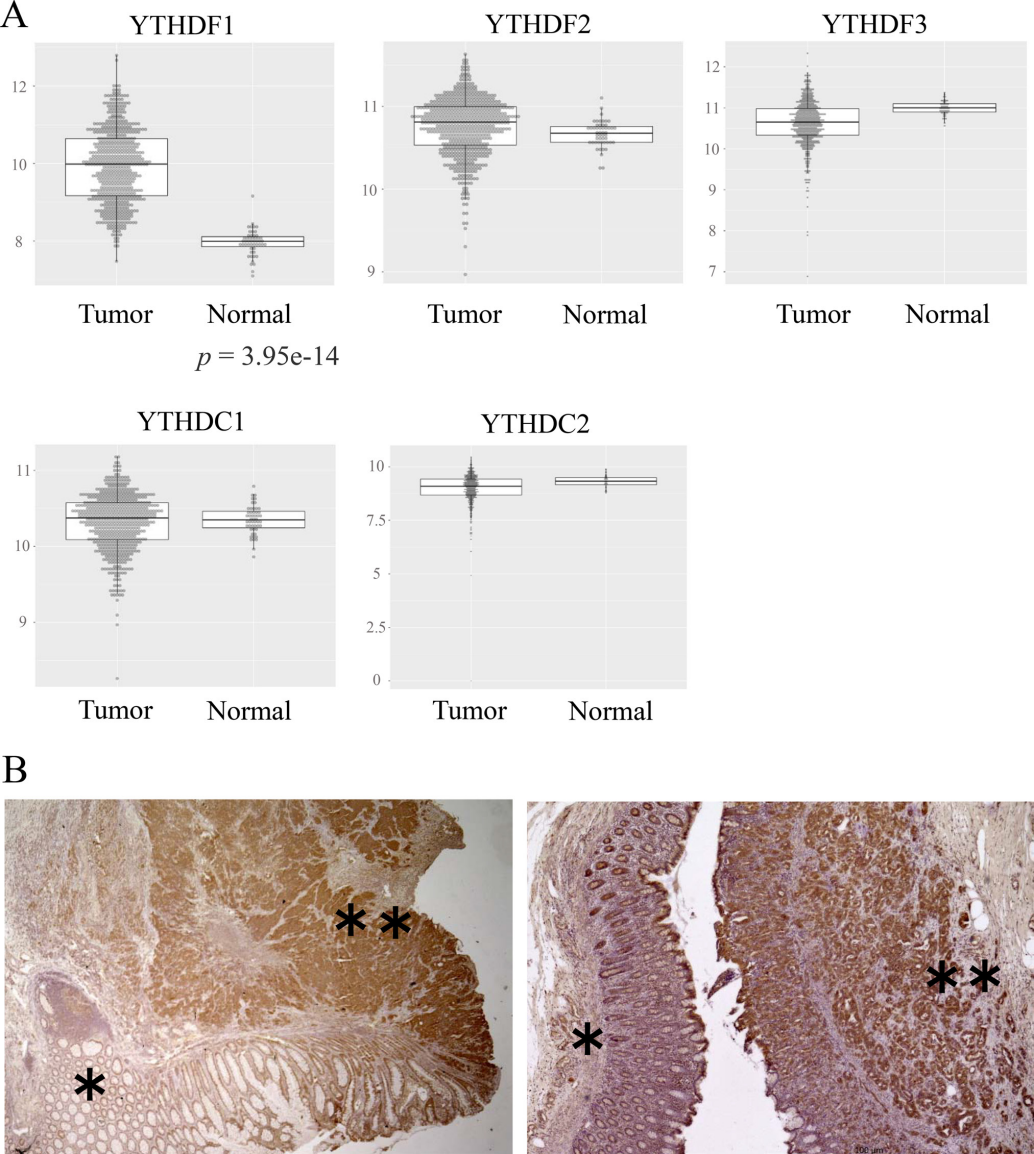
Predominant expression of YTHDF1 in CRC tissues (**A**) Expressions of the YTH domain family in colorectal tumor or normal tissue. We analyzed expressions of the YTH domain family using the TCGA colorectal adenocarcinoma (COADREAD) database, which consisted of 671 tumor parts and 51 normal tissues. (**B**) Representative immunohistochemical staining of YTHDF1 in CRC. “^*^” shows a normal part and “^**^” shows a tumor of colorectal tissue.

The study of YTH domain family expression indicated that YTHDF1 was overexpressed in CRC portions compared with normal counterparts, while other YTH domain family members, YTHDF2, 3 and YTHDC1, 2, showed little difference between cancer and normal portions in the TCGA database (https://cancergenome.nih.gov). This suggests that YTHDF1 plays a major role in CRC (Figure 1A).

To clarify the clinicopathological significance of YTHDF1, we performed immunohistochemical (IHC) staining of YTHDF1. To the best of our knowledge, the present study is the first to report on Ythdf1 protein expression in human CRC tissues. YTHDF1 was known to be involved in mRNA translation, predominantly in cytoplasm, and testis tissue was used as a positive control for the protein study [14, 15]. We confirmed that YTHDF1 is expressed in cytoplasm (Supplementary Figure 1). We performed IHC staining of YTHDF1 in CRC tissues. The result indicated that, compatible with the TCGA database study, the expression of Ythdf1 was more strongly stained in tumor tissue than in normal tissue (Figure 1B).

### High Ythdf1 expression is related to poor prognosis in overall survival

To study the role of Ythdf1 in the malignant behavior of cancer in patients, we classified patients with CRC into four groups by Ythdf1 staining intensity. Negative cytoplasm staining was score zero (0). Weak cytoplasm staining compared to the positive control was score (1+). Staining similar to the positive control was score (2+). Staining that was stronger than the positive control was score (3+). Then, we defined a score of 0 or 1+ as the Ythdf1 negative group and a score of 2+ or 3+ as the Ythdf1 positive group (Supplementary Figure 2).

**Figure 2:**
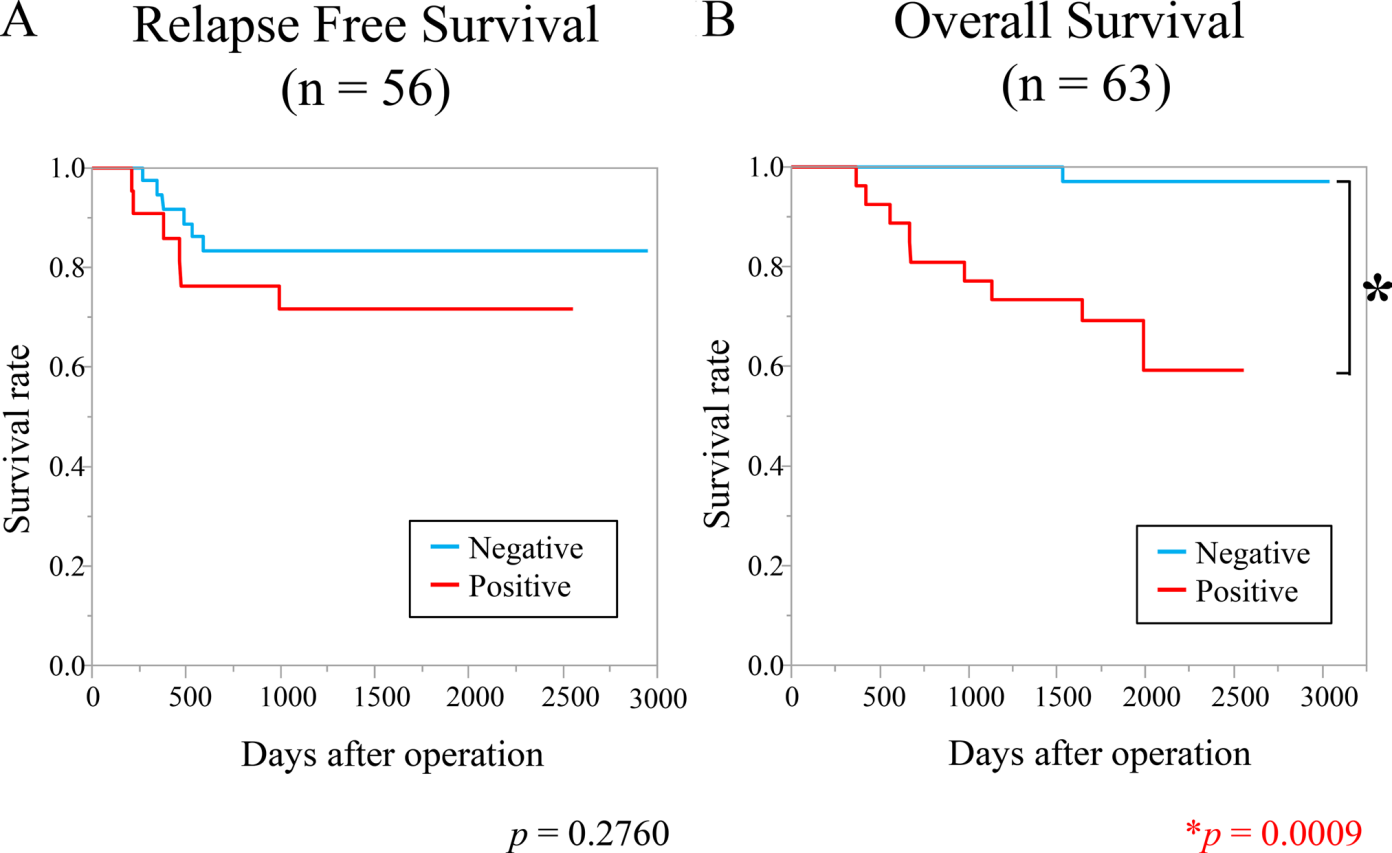
High expression of YTHDF1 was associated with poorer overall survival (**A**) Kaplan–Meier relapse-free survival curves of patients with CRC (*n* = 56). The relapse-free survival rate of patients in the YTHDF1-positive group was not significantly lower than that of patients in the negative group (*p* = 0.2760). (**B**) Kaplan–Meier overall survival curves of patients with CRC (*n* = 63) according to immunohistochemical staining of YTHDF1. The survival rate of the YTHDF1-positive group was significantly lower than that of the negative group (*p* = 0.0009).

A clinicopathological analysis showed that high Ythdf1 expression was related to tumor malignant characteristics, such as depth, lymph node metastasis, distant metastasis, and a poorer cancer stage (Table 1). We then examined whether the expression of Ythdf1 was related to survival in patients with CRC. Relapse-free survival (*n* = 56) was not significantly different between the two groups (Figure 2A). On the other hand, the Kaplan–Meier curve of overall survival (*n* = 63) showed that the positive Ythdf1 group had a significantly poorer prognosis than the negative group (Figure 2B). The multivariate analysis indicated that the Ythdf1 expression is an independent prognostic factor for overall survival of patients with CRC (Table 2).

**Table 1:** YTHDF1 expression and clinicopathological features of CRC

	Positive (*N* = 27)	Negative (*N* = 36)	*p* value
Patient background Gender (Male/Female)	18/9	23/13	1.0000
Tumor characteristics Histological type tub1/tub2/pap	1/23 /0	19/13/1	1.0000
por/muc	3/0	0/3	
Depth			0.0009^*^
Tis/T1/T2	0/1/4	8/6/8	
T3/T4	19/3	13/1	
Lymph node metastasis			0.044^*^
N0	10	23	
N1/N2/N3	11/2/4	10/1/2	
Distant metastasis			0.036^*^
M0	21	35	
M1	6	1	
Lymphatic duct invasion			0.0608
ly0	5	15	
ly1/ly2	16/6	17/4	
Venous invasion			0.3654
v0	23	27	
v1/v2	2/2	8/1	
Stage			0.0226^*^
0/I/II	0/4/5	8/12/3	
IIIa/IIIb/IV	9/3/6	8/4/1	

tub1: well-differentiated tubular adenocarcinoma, tub2: moderately differentiated tubular adenocarcinoma, pap: papillary adenocarcinoma, por: poorly differentiated adenocarcinoma, muc: mucinous adenocarcinoma, ^*^p < 0.05.

**Table 2:** Univariate and multivariate analysis for overall survival (Cox regression model)

Clinicopathological factors	Univariate			Multivariate		
	HR	95% CI	*p* value	HR	95% CI	*p* value
Gender (Male/Female)	0.78	0.22‒3.04	0.7000			
Histological type (muc, por/tub1, tub2,pap)	5.12	1.09‒18.6	0.0395^*^	18.37	2.73–184.5	0.0038^*^
Depth (Tis, T1, T2/T3, T4)	8.09	1.52‒149.1	0.0106^*^	2.37	0.26–55.8	0.470
Lymph node metastasis (positive/negative)	5.32	1.33‒35.3	0.0165^*^	2.033	0.34–20.7	0.451
Distant metastasis (positive/negative)	19.68	5.13‒80.6	< 0.0001^*^	15.00	2.90–110.4	0.0013^*^
YTHDF1 expression (positive/negative)	15.36	2.86‒284.1	0.0005^*^	9.679	1.52–194.6	0.013^*^

HR: Hazard ratio, CI: Confidence interval

^*^p < 0.05.

### Ythdf1 was associated with proliferation and chemosensitivity

**Figure 3:**
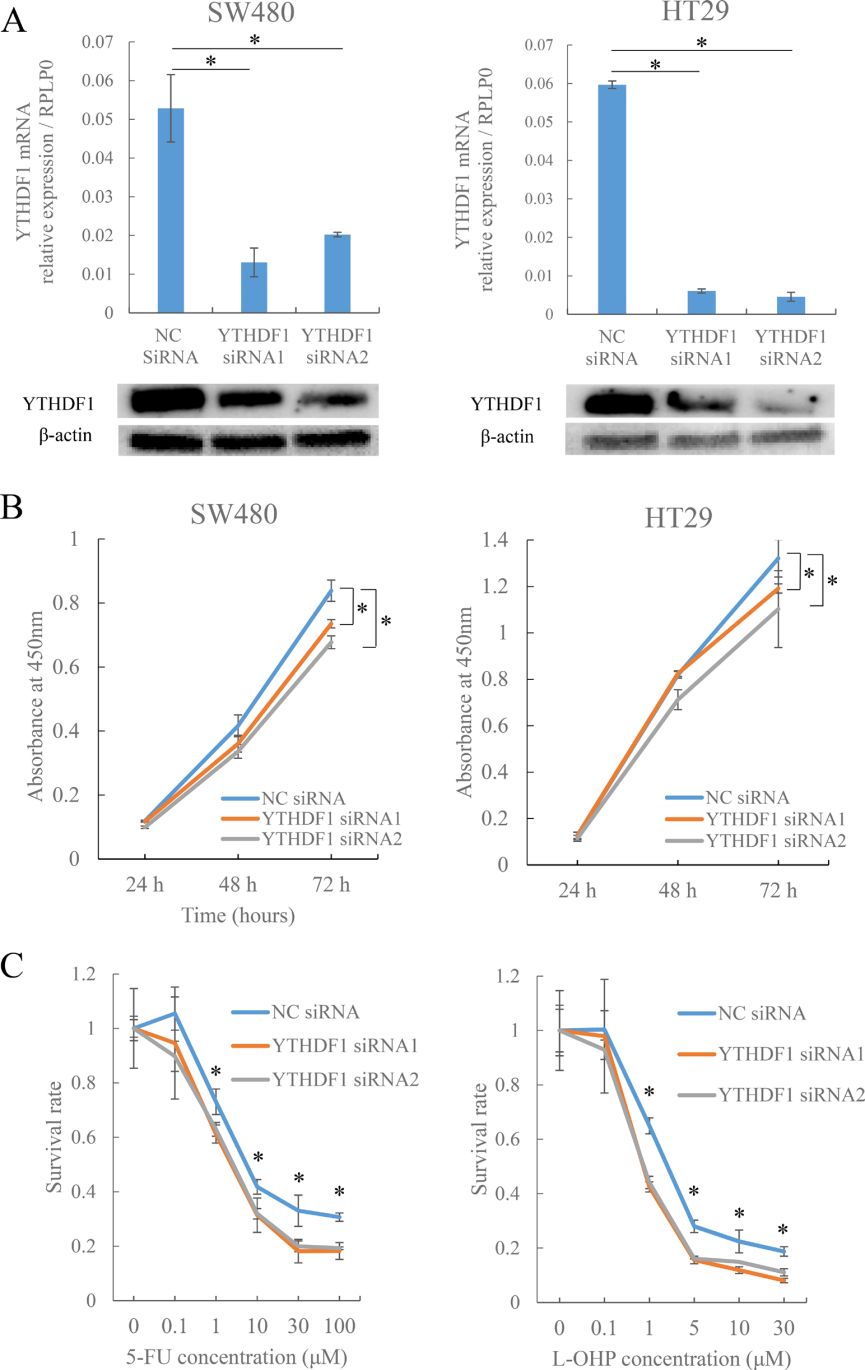
Knockdown of YTHDF1 affects cell proliferation and chemosensitivity in CRC cells (**A**) mRNA and protein expression levels of YTHDF1 compared to negative control siRNA (NC siRNA) or two types of YTHDF1 siRNA (YTHDF1 siRNA1 and siRNA2) transfected SW480 and HT29 cells. YTHDF1 expression was normalized to RPLP0 in qRT-PCR and normalized to β-actin in Western blotting. ^*^p < 0.05. (**B**) Proliferation assay after transfecting SW480 and HT29 cells with NC siRNA or YTHDF1 siRNA1 or siRNA2. Each bar represents the mean ± SEM of samples measured in triplicate. ^*^p < 0.05. (**C**) Chemosensitivity assay using 5-FU or L-OHP after transfecting HT29 cells with NC siRNA or YTHDF1 siRNA1 or siRNA2. Each bar represents the mean ± SEM of samples measured in triplicate. ^*^p < 0.05.

This study indicated that Ythdf1 was highly expressed in tumors and was relevant in the prognosis of patients with CRC. As we are interested in how the Ythdf1 protein is involved in the malignant behavior of CRC, we performed an *in vitro* study. We first studied YTHDF1 mRNA expression in CRC cell lines (Supplementary Figure 3). The data indicated that SW480 and HT29 cells had relatively higher expression in YTHDF1, thus, we used SW480 and HT29 cells for the knockdown experiment of YTHDF1 by siRNA. We confirmed that the expression was reduced compared with the control in mRNA and protein levels (Figure 3A).

Next, we performed a proliferation assay with the knockdown cells, which showed that the knockdown of YTHDF1 resulted in the inhibition of cell proliferation compared with cells transfected as a negative control siRNA in both SW480 and HT29 cells (Figure 3B).

Moreover, we performed a chemosensitivity assay using fluorouracil (5-FU) and oxaliplatin (L-OHP), which are anticancer drugs commonly used in the therapeutic treatment of patients with CRC [16]. The results from the chemosensitivity assay showed that transfected YTHDF1 siRNA cells had a higher sensitivity to both 5-FU and L-OHP than cells transfected with negative control siRNA in HT29 cells (Figure 3C). The results of value of 50% inhibitory concentration (IC50) is shown in Supplementary Table 1.

### YTHDF1 was transcriptionally regulated by c-Myc in CRC

**Figure 4:**
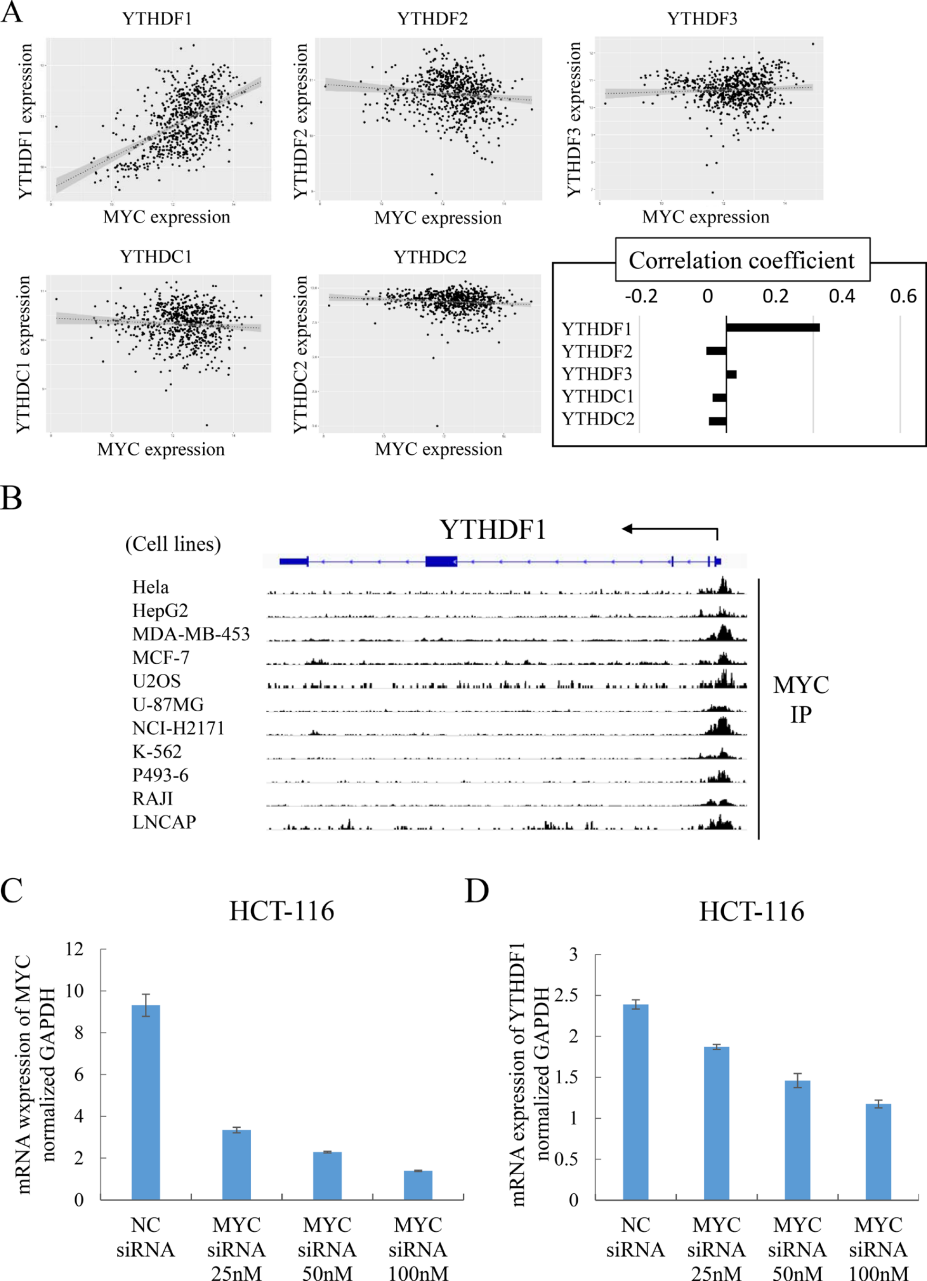
YTHDF1 was transcriptionally regulated by c-MYC (**A**) Correlations between the YTH domain family and c-MYC. We used the TCGA colorectal adenocarcinoma (COADREAD) database, which consisted of 677 tumor parts. (**B**) ChIP-atlas (http://chip-atlas.org/) data show that c-MYC binds to the transcription initiation region of YTHDF1. (**C**) mRNA expression level of c-MYC compared to negative control siRNA (NC siRNA) or c-MYC siRNA (25, 50, and 100 nM) transfected cells. YTHDF1 expression was normalized to GAPDH in qRT-PCR. ^*^p < 0.05. (**D**) mRNA expression level of YTHDF1 compared to negative control siRNA (NC siRNA) or c-MYC siRNA (25, 50, and 100 nM) transfected cells. YTHDF1 expression was normalized to GAPDH in qRT-PCR. ^*^p < 0.05.

This study demonstrated that YTHDF1 plays a role in the control of cell proliferation and chemoresistance of CRC cells, suggesting its oncogenic property. Nevertheless, how YTHDF1 works in the transcription network in cancer is still unclear. To this end, we studied the TCGA database and found that the expression of oncogenic transcription factor c-Myc was associated with the expression of YTHDF1 in CRC (R = 0.537, Figure 4A). We then studied whether c-Myc was associated with the 5′ region of the transcription start site of the YTHDF1 gene by chromatin immunoprecipitation database (ChIP-atlas) in cancer cells lines. The data indicated that c-Myc was bound to the 5′ region of the YTHDF1 gene (Figure 4B), suggesting the role of c-Myc in the control of Ythdf1. We studied the other family (YTHDF1, 2, 3 and YTHDC1, 2) by utilizing the ChIP-atlas database, confirming a role of c-Myc in YTHDF1 (Supplementary Figure 4). To confirm whether c-Myc plays a biological role in cancer cells, we then performed a knockdown experiment of c-Myc. The results indicated that c-Myc knockdown resulted in the inhibition of the transcription target YTHDF1 in a dose-dependent manner of transfection. The expression analysis of c-Myc knockdown showed no significant alterations in the other family, confirming a role of c-Myc in YTHDF1 (Supplementary Figure 5). Taken together, this study indicated that oncogene c-Myc promotes YTHDF1 expression in CRC cells.

## DISCUSSION

Here, we showed that (1) the epitranscriptome m^6^A reader YTHDF1 was overexpressed among the YTH domain family and the Ythdf1 protein was expressed at a higher level in cancer portions than in non-cancerous normal portions in the same patients based on immunohistochemistry; (2) the study of clinicopathological parameters indicated that cases with high expression of the Ythdf1 protein were associated with malignant phenotypes and they had a poor CRC prognosis; (3) the multivariate analysis demonstrated that the YTHDF1 expression is an independent prognostic factor of patients with CRC; (4) the *in vitro* study indicated the role of YTHDF1 in chemosensitivity; (5) the oncogene c-MYC promotes YTHDF1 expression in CRC. The present study supports the notion that the c-MYC driving transcription network is involved in the malignant behavior of CRC cells at least partially via the function of Ythdf1. Recent studies indicated that high expression of Wilms’ tumor 1-associating protein (Wtap), a component of the RNA methylases Mettl3-Mettl14-Wtap complex, was correlated with poor postoperative survival in patients with glioma [17], that the eraser, fat mass- and obesity-associated (Fto), plays a role in decreased m^6^A in peripheral blood RNA from diabetic patients [18], and that another eraser, α-ketoglutarate-dependent dioxygenase alkB homolog 5 (Alkbh5) protein, maintains tumorigenicity of glioma stem-like cells [19] and modulates breast cancer cell in hypoxia [20], suggesting that m^6^A modification is a critical marker in not only cancer but also other metabolic diseases such as diabetes. To the best of our knowledge, this study is the first to report on the role of YTHDF1 in CRC. Given that RNA m^6^A methylation reportedly regulates ultraviolet-induced DNA damage response, m^6^A methylation may be critical for the preservation of genomic integrity, the mechanism function fundamental in early and advanced cancer stages [21]. Indeed, the present study indicated that the knockdown experiment of YTHDF1 by siRNA resulted in the sensitization of cancer cells to the exposure of 5-FU and L-OHP, suggesting a common fundamental mechanism might be involved in the YTHDF1 pathway, such as m^6^A dependent translation, regardless of cancer tissue types, though further study would be necessary. Taken together with the present observation of YTHDF1 role in cancer cell proliferation, the data suggest the candidacy of YTHDF1 as a possible therapeutic target.

Importantly, in this study we found that oncogene c-Myc activates the transcriptional expression on the YTHDF1 gene. A chromatin immunoprecipitation study supports the notion that c-Myc modulates the activation of YTHDF1 in the 5′-region of the transcription start site. The expression study indicated that c-Myc has a correlation with YTHDF1, but not with other family, though we found the c-Myc binding site(s) around the transcription initiation regions of those family genes through the study of chromatin immunoprecipitation database (ChIP-atlas) in cancer cells. Then, the c-Myc knockdown experiment indicated that the expression was altered in YTHDF1, but not with other family, suggesting a critical role on c-Myc in YTHDF1 expression. Recently, numerous studies indicated a critical role of c-Myc in inflammatory and cancerous colorectal diseases [22], at least through the involvement of the Wnt/β-catenin signaling pathway [23]. Considering that c-Myc is overexpressed in 70% cases with CRC and generates unique transcription networks, which characterizes cancer [24, 25], this study suggests that c-Myc induces an oncogenic effect on the transactivation of the m^6^A reader YTHDF1 in an uncharacterized pathway.

As to the control of YTHDF1 expression, other factors such as miRNAs remains to be understood perfectly. Considering that the present study indicated that YTHDF1 is involved in malignant nature of cancer, we propose that a group of miRNAs which targeting on 3′ untranslated region might play a role in the tumor suppressor function. Previous studies indicated that m^6^A is the most prevalent type among various RNA modifications, such as m^5^C, hm^5^C, m^6^A, and m^1^A and suggested their eligibility as therapeutic targets in cancer [9]. Given that m^6^A modulates translation efficiency [15], we propose that the overexpressed Ythdf1 gene may be involved in the induction of downstream protein synthesis in an eIF complex on a ribosome of unidentified targets associated with therapeutic resistance. Although downstream targets of YTHDF1 need to be elucidated in future studies, our findings clearly indicated that epigenetic regulation by YTHDF1 plays an important role in cancer progression processes in CRC.

## MATERIALS AND METHODS

### Clinical tissue samples

CRC tissue samples (*n* = 63) were collected during surgery between 2007 and 2008 at the Department of Gastroenterological Surgery, Osaka University. All patients were clearly diagnosed with CRC based on the clinicopathological criteria described by the Japanese Society for Cancer of the Colon and Rectum. None of the patients had undergone preoperative chemotherapy or irradiation. Samples were fixed in buffered formalin at 4°C overnight, processed through graded ethanol solutions, and embedded in paraffin. The specimens were appropriately used under the approval of the Ethics Committee at the Graduate School of Medicine, Osaka University.

### Immunohistochemical staining

Immunohistochemical staining was performed as previously described [24]. The anti-Ythdf1 rabbit antibody (17479-1-AP; Proteintech, IL, USA) was used for immunohistochemical staining, and the slides were incubated overnight at 4°C with the antibody at a 1:200 dilution. We used normal testis as a positive YTHDF1 control according to the package insert of the YTHDF1 antibody. We assigned an intensity score of 2+ to cytoplasm stained as intensely as the positive control, whereas unstained cytoplasm was assigned a score of 0. Cytoplasm that was stained stronger or weaker than the positive control was assigned scores of 3+ or 1+, respectively. In the following analysis, we defined scores of 0 or 1+ as the YTHDF1-negative group, and scores of 2+ or 3+ as the YTHDF1-positive group (Supplementary Figure 2). The staining was reviewed by three independent pathologists without knowledge of patient outcomes.

### Cell lines and cell culture

Human CRC cell lines SW480, CaCO2, HT29, RKO, DLD-1, KM12SM, HCT-116, and LoVo were purchased from the American Type Culture Collection (Manassas, VA, USA) and maintained in Dulbecco’s modified Eagle’s medium (Sigma-Aldrich, St. Louis, MO, USA) supplemented with 10% FBS at 37°C at 5% CO_2_ in a humidified incubator.

### RNA interference

Two types of YTHDF1-specific small interfering RNAs (siRNA; Sigma-Aldrich) were used to knock down YTHDF1 messenger RNA (mRNA). YTHDF1, or the negative control siRNA, was transfected into SW480 or HT29 cells at a 50 nM final concentration with lipofectamine RNAiMax (Thermo Fisher Scientific, Yokohama, Japan) according to the manufacturer’s protocol. RNA was extracted 72 h after transfection. The sequence of siRNA against YTHDF1 was as follows:

YTHDF1 siRNA1 5′-CCUACGGACAGCUCAGUAATT-3′; YTHDF1 siRNA2 5′-CCUGCUCUUCAGCGUCAAUTT-3′.

### Real-time quantitative reverse transcriptase-polymerase chain reaction (qPCR)

Total RNA was extracted from cultured cells using TRIzol^®^ RNA Isolation Reagents (Thermo Fisher Scientific) as previously described [26]. The RNA quality was checked (RNA concentration > 0.5 μg/μL and OD260/280 = 1.8–2.0). cDNA was synthesized from 10 ng of total RNA using the High Capacity RNA-to-cDNA Kit (Thermo Fisher Scientific) according to the manufacturer’s protocol. PCR was performed in a Light Cycler™ 2.0 System (Roche Applied Science, Tokyo, Japan) using the Thunderbird^®^ SYBR^®^ qPCR mix (Toyobo Life Science, Osaka, Japan). Relative expression was calculated by the CT-based calibrated standard curve method. The calculated values were then normalized against the expression of GAPDH or RPLP0 for mRNA. Since we noted that the RPLP0 data vary in the CT value of PCR in siRNA knockdown experiment of c-Myc, we used the data of GAPDH in the experiment. Each independent experiment was performed from independently obtained samples as principle three times, to confirm the reproducible data.

### The following primers were used

*YTHDF1*, 5′-ATGTCGGCCACCAGCGTGGACA-3′ (forward) and 5′-TCATTGTTTGTTTCGACTCTGC-3′ (reverse); *GAPDH*, 5′-AGCCACATCGCTCAGACAC-3′ (forward) and 5′-GCCCAATACGACCAAATCC-3′ (reverse); *RPLP0*, 5′-AGCCACATCGCTCAGACAC-3′ (forward) and 5′-GCCCAATACGACCAAATCC-3′ (reverse).

### Western blot analysis

Total protein (20 μg) was extracted from cultured cells using a radioimmunoprecipitation assay

buffer containing a protease inhibitor and a phosphatase inhibitor (Thermo Fisher Scientific). The protein was electrophoresed on 10% sodium dodecyl sulfate polyacrylamide gel electrophoresis gels and electroblotted onto polyvinylidene fluoride membranes (Merck Millipore, Darmstadt, Germany) at 150 V for 60 min. β-actin antibodies (A2066, Sigma-Aldrich) were used as a loading control. After blocking with 5% skim milk for 1 h, these membranes were incubated with primary antibodies at the appropriate concentrations (1:1,000 for YTHDF1 and 1:4,000 for β-actin) at 4°C overnight. After incubating with secondary antibodies, the protein bands were detected using the Amersham ECL Detection System (Amersham Biosciences, Piscataway, NJ, USA). Each independent experiment was performed from independently obtained samples as principle at least three times, to confirm the reproducible data.

### Proliferation assay

Cells were seeded at a density of 4 × 10^3^ cells per well in 96-well plates. Cell proliferation was assessed 24–72 h after transfection at intervals of 24 h using the Cell Counting Kit-8 (Dojindo, Tokyo, Japan) according to the manufacturer’s protocol. We confirmed that the absorbance in was well compatible with the manual methods of cell counting, before starting experiments.

### Chemosensitivity assay

HT29 cell lines were seeded at a density of 4 × 10^3^ cells per well in 96-well plates and precultured for 24 h. They were then exposed to various concentrations of 5-FU (Tokyo Chemical Industry, Tokyo, Japan) and L-OHP (Yakult, Tokyo, Japan) for 72 h. The *in vitro* cytotoxic effects of 5-FU and L-OHP were evaluated by the Cell Counting Kit-8 according to the manufacturer’s protocol. The IC_50_ value is shown in the Supplementary Table 1.

### Data analysis

The database in the ChIP-atlas (http://chip-atlas.org/) was used for the analysis. The peak browser in the ChIP-atlas allowed the identification of binding site(s) around the transcription start site of genes.

### Statistical analysis

Statistically significant differences were determined by Student’s *t*-test and Fisher’s exact probability test, as appropriate. JMP^® Pro^12 (SAS Institute Inc., Cary, NC, USA) was used for all statistical analyses. In the Kaplan–Meier method, we included all patients (*n* = 63) in the overall survival analysis. Seven patients with an unresectable distant metastasis were excluded from the relapse-free survival analysis. The log-rank test was used to check differences between the survival curves. Variables shown by univariate analysis to be significantly correlated with survival were entered into a Cox proportional hazards regression model for multivariate analysis. Data are reported as means ± SEM. Results were considered statistically significant when *p* < 0.05 was obtained.

## SUPPLEMENTARY MATERIALS FIGURES AND TABLE



## References

[1] Torre LA, Bray F, Siegel RL, Ferlay J, Lortet-Tieulent J, Jemal A (2015). Global cancer statistics, 2012. CA Cancer J Clin.

[2] Kim TM, Lee SH, Chung YJ (2013). Clinical applications of next-generation sequencing in colorectal cancers. World J Gastroenterol.

[3] Murakami K, Matsubara H (2017 Aug 9). Chronology of gastrointestinal cancer. Surg Today.

[4] Goldberg AD, Allis CD, Bernstein E (2007). Epigenetics: a landscape takes shape. Cell.

[5] Träger MM, Dhayat SA (2017). Epigenetics of epithelial-to-mesenchymal transition in pancreatic carcinoma. Int J Cancer.

[6] Desrosiers R, Friderici K, Rottman F (1974). Identification of methylated nucleosides in messenger RNA from Novikoff hepatoma cells. Proc Natl Acad Sci U S A.

[7] Maity A, Das B (2016). N6-methyladenosine modification in mRNA: machinery, function and implications for health and diseases. FEBS J.

[8] Batista PJ (2017). The RNA Modification N6-methyladenosine and Its Implications in Human Disease. Genomics Proteomics Bioinformatics.

[9] Esteller M, Pandolfi PP (2017). The Epitranscriptome of Noncoding RNAs in Cancer. Cancer Discov.

[10] Fustin JM, Doi M, Yamaguchi Y, Hida H, Nishimura S, Yoshida M, Isagawa T, Morioka MS, Kakeya H, Manabe I, Okamura H (2013). RNA-methylation-dependent RNA processing controls the speed of the circadian clock. Cell.

[11] Bodi Z, Bottley A, Archer N, May ST, Fray RG (2015). Yeast m6A Methylated mRNAs Are Enriched on Translating Ribosomes during Meiosis, and under Rapamycin Treatment. PLoS One.

[12] Xu K, Yang Y, Feng GH, Sun BF, Chen JQ, Li YF, Chen YS, Zhang XX, Wang CX, Jiang LY, Liu C, Zhang ZY, Wang XJ (2017). Mettl3-mediated m6A regulates spermatogonial differentiation and meiosis initiation. Cell Res.

[13] Guo M, Liu X, Zheng X, Huang Y, Chen X (2017). m6A RNA Modification determines cell fate by regulating mRNA degradation. Cell Reprogram.

[14] Xu C, Liu K, Ahmed H, Loppnau P, Schapira M, Min J (2015). Structural basis for the discriminative recognition of N6-methyladenosine RNA by the human YT521-B homology domain family of proteins. J Biol Chem.

[15] Wang X, Zhao BS, Roundtree IA, Lu Z, Han D, Ma H, Weng X, Chen K, Shi H, He C (2015). N(6)-methyladenosine Modulates Messenger RNA Translation Efficiency. Cell.

[16] Colvin H, Mizushima T, Eguchi H, Takiguchi S, Doki Y, Mori M (2017). Gastroenterological surgery in Japan: The past, the present and the future. Ann Gastroenterol Surg.

[17] Xi Z, Xue Y, Zheng J, Liu X, Ma J, Liu Y (2016). WTAP expression predicts poor prognosis in malignant glioma patients. J Mol Neurosci.

[18] Shen F, Huang W, Huang JT, Xiong J, Yang Y, Wu K, Jia GF, Chen J, Feng YQ, Yuan BF, Liu SM (2015). Decreased N(6)-methyladenosine in peripheral blood RNA from diabetic patients is associated with FTO expression rather than ALKBH5. J Clin Endocrinol Metab.

[19] Zhang S, Zhao BS, Zhou A, Lin K, Zheng S, Lu Z, Chen Y, Sulman EP, Xie K, Bögler O, Majumder S, He C, Huang S (2017). m6A demethylase ALKBH5 maintains tumorigenicity of glioblastoma stem-like cells by sustaining FOXM1 expression and cell proliferation program. Cancer Cell.

[20] Zhang C, Zhi WI, Lu H, Samanta D, Chen I, Gabrielson E, Semenza GL (2016). Hypoxia-inducible factors regulate pluripotency factor expression by ZNF217- and ALKBH5-mediated modulation of RNA methylation in breast cancer cells. Oncotarget.

[21] Xiang Y, Laurent B, Hsu CH, Nachtergaele S, Lu Z, Sheng W, Xu C, Chen H, Ouyang J, Wang S, Ling D, Hsu PH, Zou L (2017). RNA m6A methylation regulates the ultraviolet-induced DNA damage response. Nature.

[22] Sipos F, Firneisz G, Műzes G (2016). Therapeutic aspects of c-MYC signaling in inflammatory and cancerous colonic diseases. World J Gastroenterol.

[23] Yan M, Li G, An J (2017). Discovery of small molecule inhibitors of the Wnt/β-catenin signaling pathway by targeting β-catenin/Tcf4 interactions. Exp Biol Med (Maywood).

[24] Rennoll S, Yochum G (2015). Regulation of MYC gene expression by aberrant Wnt/β-catenin signaling in colorectal cancer. World J Biol Chem.

[25] Hamabe A, Konno M, Tanuma N, Shima H, Tsunekuni K, Kawamoto K, Nishida N, Koseki J, Mimori K, Gotoh N, Yamamoto H, Doki Y, Mori M (2014). Role of pyruvate kinase M2 in transcriptional regulation leading to epithelial–mesenchymal transition. Proc Natl Acad Sci U S A.

[26] Sugimura K, Fujiwara Y, Omori T, Motoori M, Miyoshi N, Akita H, Gotoh K, Kobayashi S, Takahashi H, Noura S, Ohue M, Yamamoto T, Sakon M (2016). Clinical importance of a transcription reverse-transcription concerted (TRC) diagnosis using peritoneal lavage fluids obtained pre- and post-lymphadenectomy from gastric cancer patients. Surg Today.

