# Supplementary material for: Oncogene c-Myc promotes epitranscriptome m6A reader YTHDF1 expression in colorectal cancer
Source: Oncotarget. 2017 Dec 21;9(7):7476–86. doi: 10.18632/oncotarget.23554 (PMC5800917; doi:10.18632/oncotarget.23554)
Supplement: Supplementary file 1 [file oncotarget-09-7476-s001.pdf]

Oncogene c-Myc promotes epitranscriptome m^{6}A reader YTHDF1 expression in colorectal cancer

SUPPLEMENTARY MATERIALS

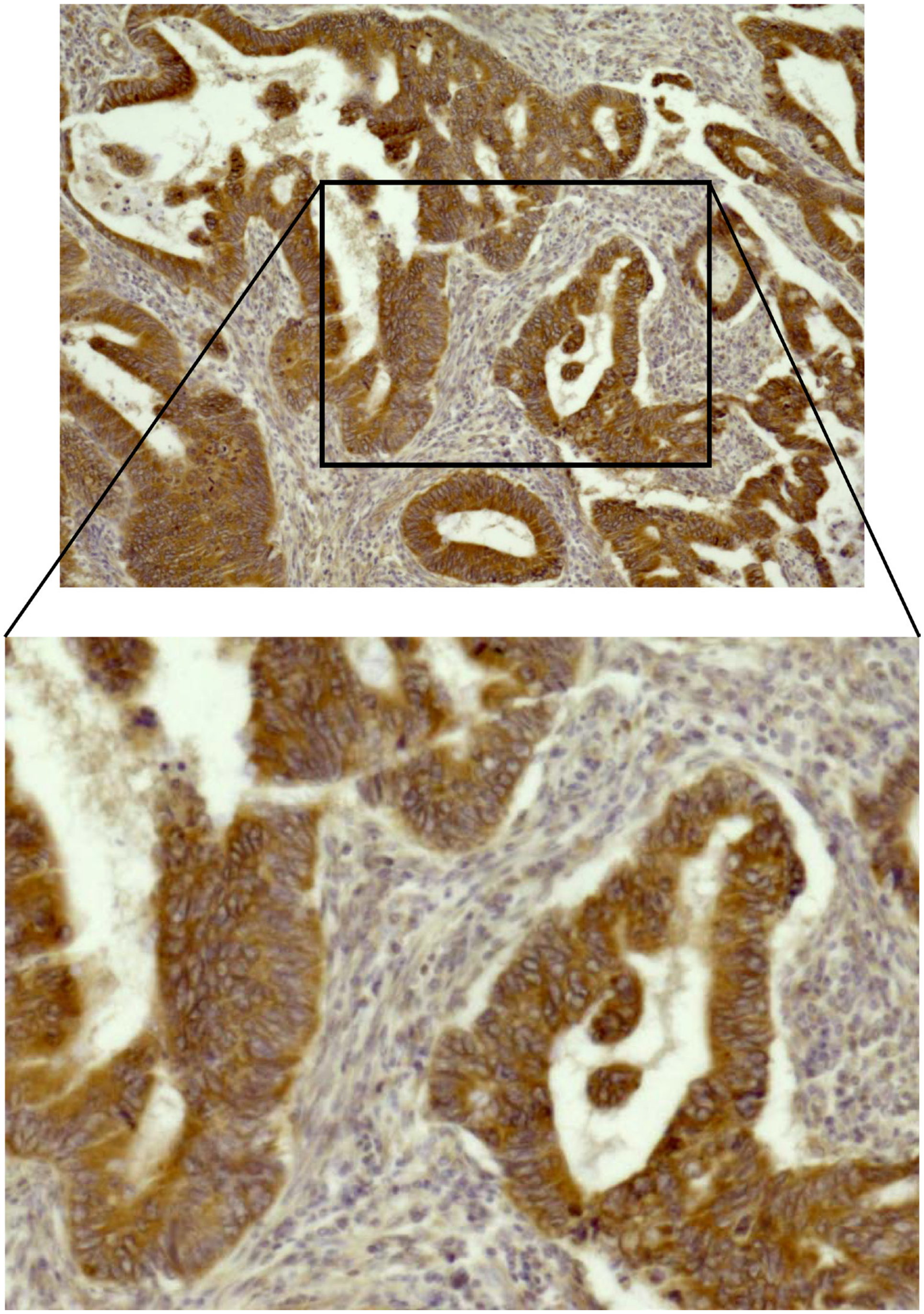

Supplementary Figure 1: Representative immunostaining of YTHDF1 in CRC. The stained part is the cytoplasm.

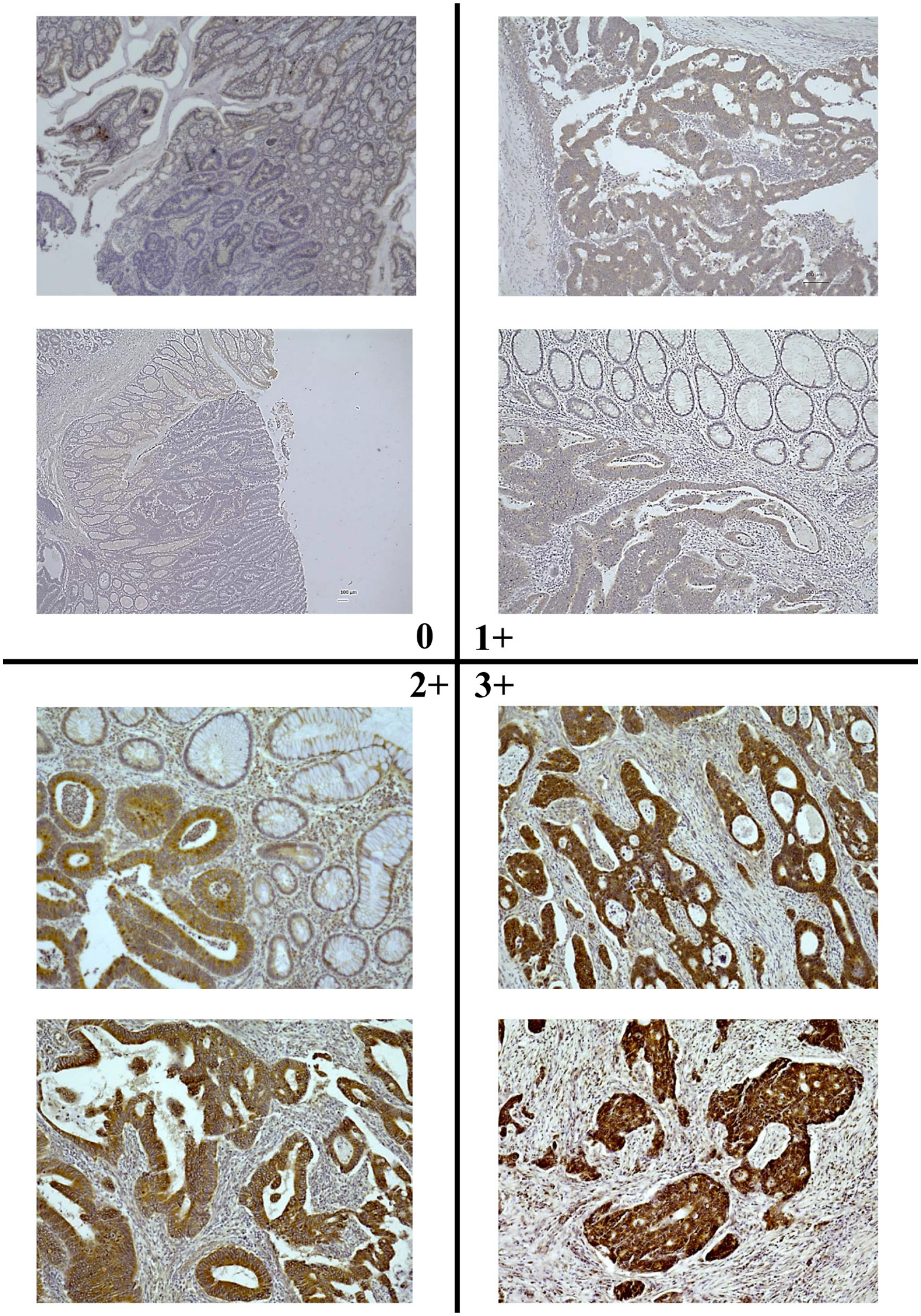

Supplementary Figure 2: Representative immunostaining score of YTHDF1 in CRC. The stained parts were assigned scores of 0, 1+, 2+, and 3+, as described in text.

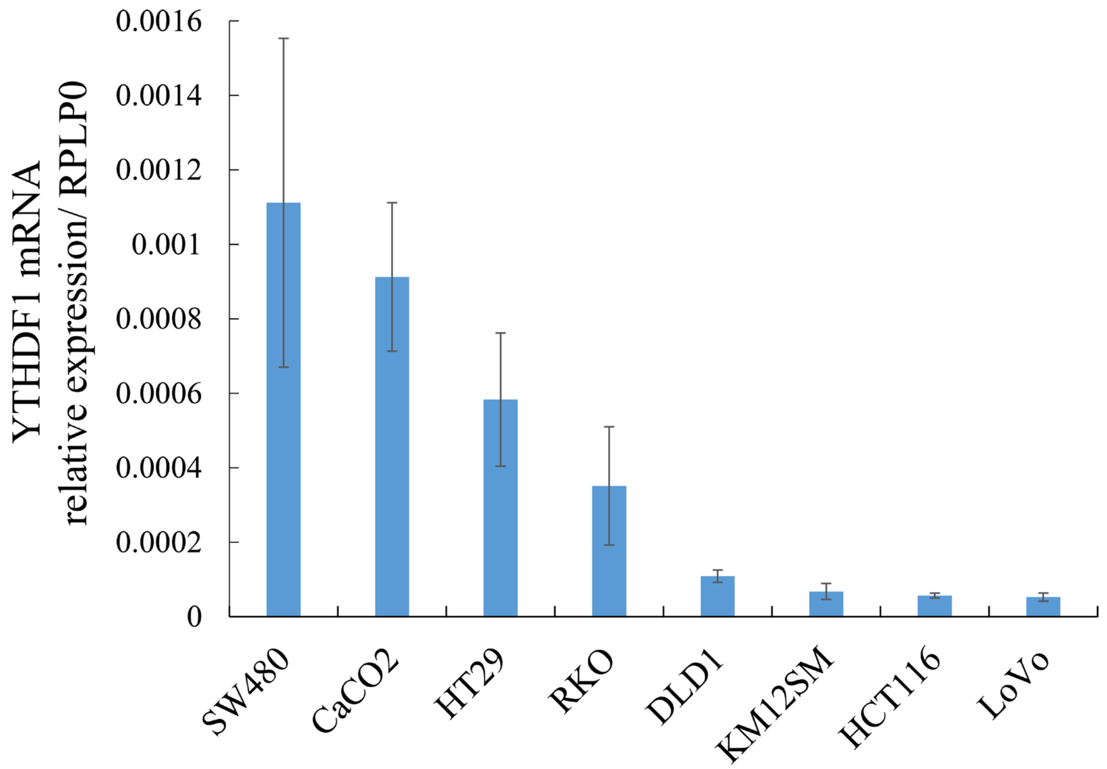

Supplementary Figure 3: YTHDF1 expression in CRC cell lines. mRNA expression levels of YTHDF1 in CRC cell lines were determined by qRT-PCR and normalized to RPLP0.

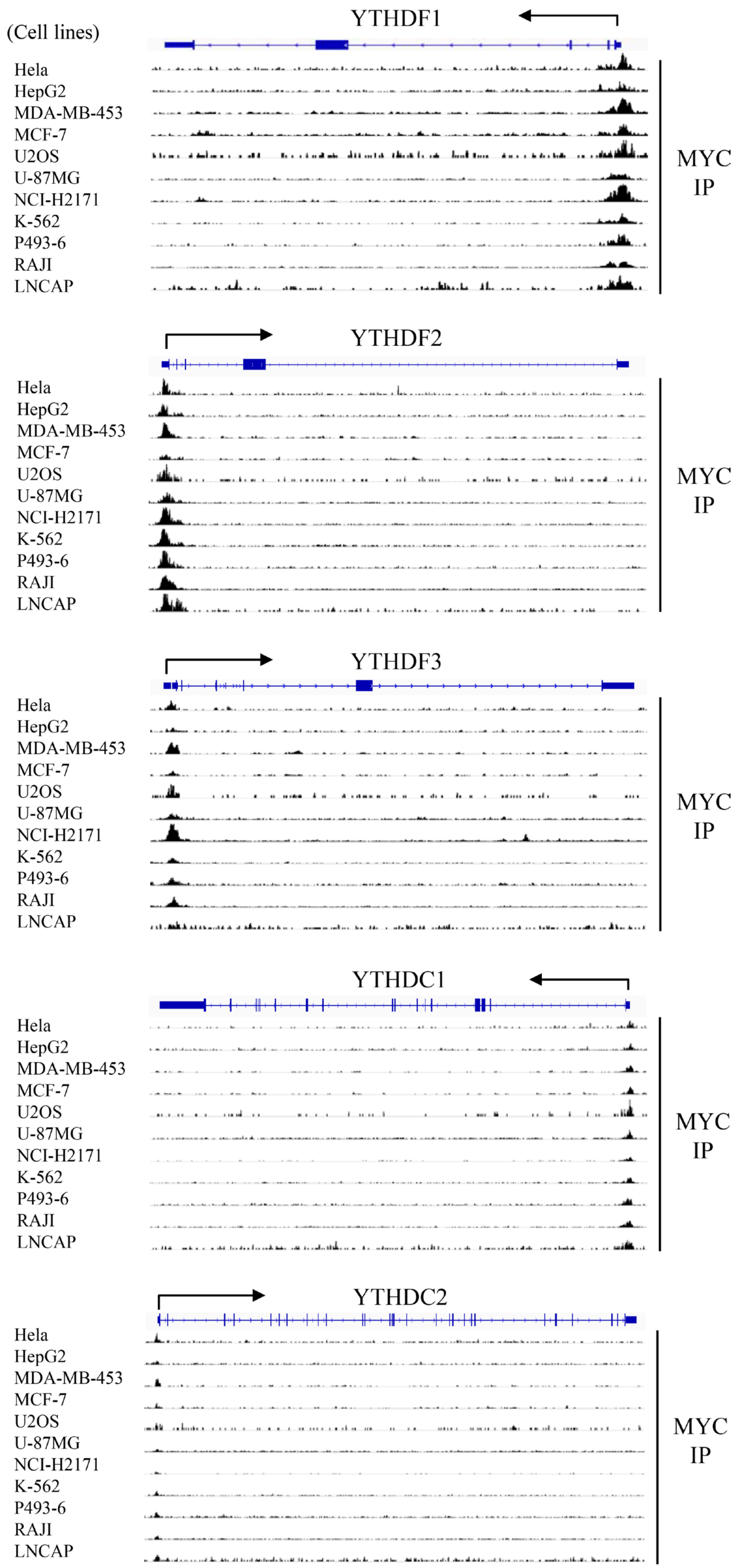

Supplementary Figure 4: c-MYC ChIP-Seq analysis of YTH domain family. The database in the ChIP-atlas (http://chip-atlas.org/) was used for the analysis. The peak browser in the ChIP-atlas showed the binding sites of c-MYC around the transcription start site of YTH domain family genes.

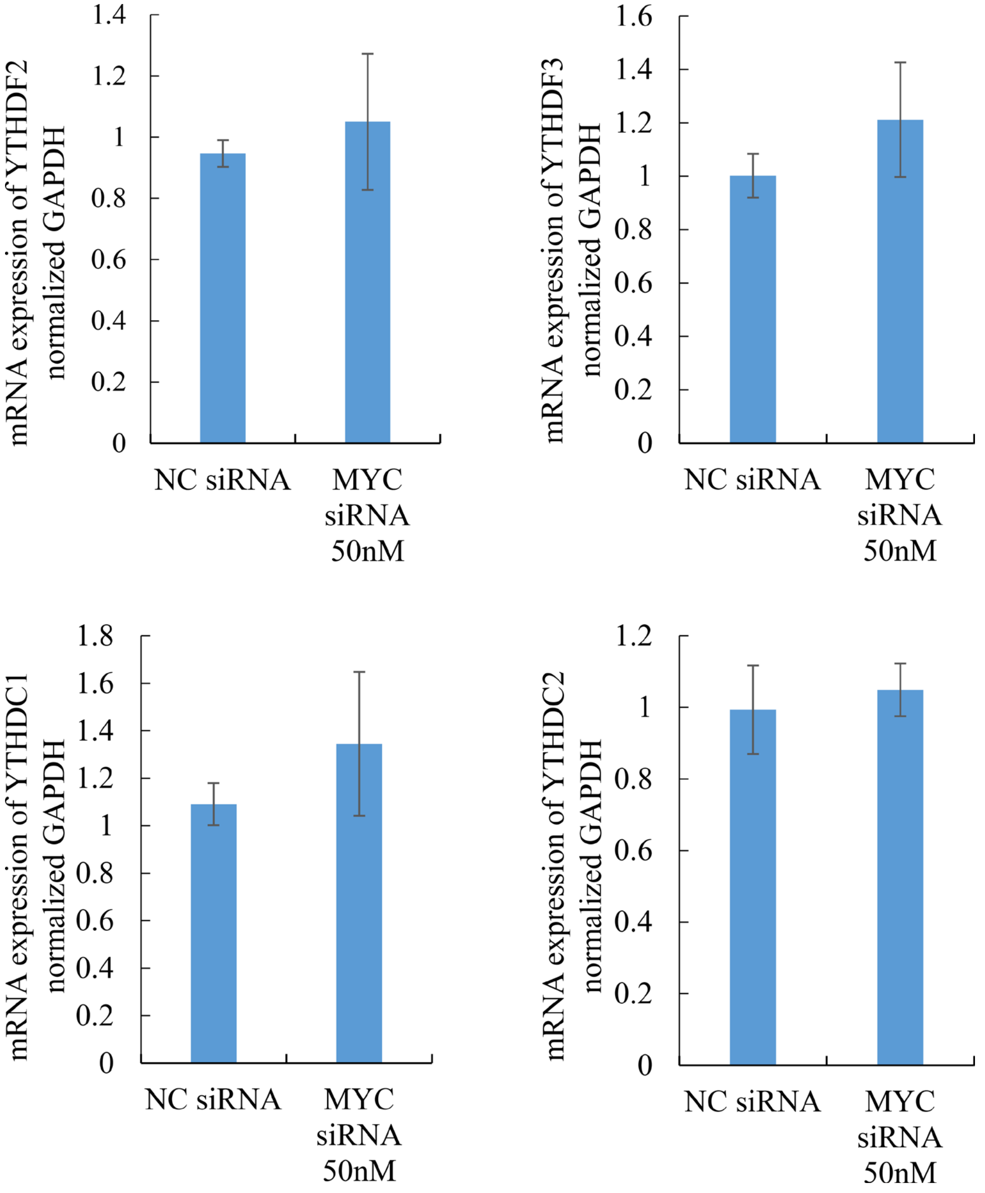

Supplementary Figure 5: Expression analysis of YTH domain family in c-Myc knockdown. The c-Myc knockdown experiment was performed in HCT-116 cells and the expression of YTH domain family genes were assessed by PCR method. In contrast to the significance in YTHDF1 (Fig. 4D), no significant changes of YTHDF2, YTHDF3, YTHDC1 and YTHDC2 expressions were detected by c-Myc knockdown.

Supplementary Table 1: IC_{50} values of 5-FU and L-OHP

	**5-FU**		**L-OHP**	
	**IC50 of 5-FU (µM)**	**Degree of resistance to 5-FU**	**IC50 of L-OHP (µM)**	**Degree of resistance to L-OHP**
NC siRNA	5.47	1	1.92	1
YTHDF1 siRNA1	2.38	0.46	0.64	0.33
YTHDF1 siRNA2	2.61	0.48	0.71	0.36

NC, negative control
